# Healthcare utilization and its association with socioeconomic status in China: Evidence from the 2011–2018 China Health and Retirement Longitudinal Study

**DOI:** 10.1371/journal.pone.0297025

**Published:** 2024-03-14

**Authors:** Xi Li, Itismita Mohanty, Peipei Chai, Theo Niyonsenga

**Affiliations:** 1 Health Research Institute (HRI), Faculty of Health, University of Canberra, Canberra, Australia; 2 Department of Health Economics and National Health Accounts Research, China National Health Development Research Center, Beijing, China; Shiraz University of Medical Sciences, ISLAMIC REPUBLIC OF IRAN

## Abstract

**Introduction:**

Healthcare utilization often favors the higher-socioeconomic status (SES) and having chronic diseases may exacerbate this inequality. This study aims to examine the trends in health service use over time, the effect of SES on healthcare utilization, and the role of chronic diseases in this association.

**Methods:**

Data used in this study were from the China Health and Retirement Longitudinal Study (CHARLS) in 2011, 2013, 2015, and 2018, which is the first nationally representative survey of the middle-aged and older. The sample included people aged 45 years and older who responded to all the waves. A total of 10,922 adults were included in this study. Healthcare utilization was categorized into outpatient and inpatient service use and SES was measured by per-capita household expenditure. A multilevel zero-inflated negative binomial regression model was performed to analyze outpatient and inpatient service use, separately.

**Results:**

The rates of outpatient service use in 2011, 2013, 2015, and 2018 were 19.11%, 21.45%, 20.12%, and 16.32%, respectively, while the rates of inpatient service use were 8.40%, 13.04%, 14.17%, and 18.79%, respectively. Compared to individuals in the lowest quintile of per-capita household expenditure, those in higher quintiles had higher odds of outpatient service use (Q2: odds ratio = 1.233, *p* < 0.0001; Q3: 1.416, *p* < 0.0001; Q4: 1.408, *p* < 0.0001; or Q5: 1.439, *p* < 0.0001) and higher rates of inpatient service use (Q2: incidence rate ratio = 1.273, *p* < 0.0001; Q3: 1.773, *p* < 0.0001; Q4: 2.071, *p* < 0.0001; or Q5: 1.992, *p* < 0.0001). Additionally, having morbidity generally increased healthcare utilization, but did not play a significant role in moderating the relationship between SES and healthcare utilization.

**Conclusions:**

Healthcare utilization rates were overall low in China, but relatively high for people in higher quintiles of per-capita household expenditure or those with morbidity, compared to their counterparts. Policy actions are required to provide more health education to the public, to further optimize health insurance schemes targeting outpatient services, especially for the low-SES, and to establish new health delivery models for NCD management in the primary health care setting.

## Introduction

Equitable access to health care is a global public health challenge, including in China [1–3]. Since the economic reform and opening-up in 1978, China has faced growing disparities in health service use [1–11]. Therefore, addressing disparities in healthcare utilization has become the priority in China’s health system [6]. Globally, one commonly adopted approach is to implement social health insurance schemes [12], as low affordability is a major obstacle to health equity [6, 13] and health insurance can enhance individual’s affordability [4, 14]. Through mobilizing funds and risk pooling, the rollout of health insurance schemes may redress the systematic differences in healthcare utilization [15].

Similarly, through the pursuit of universal health coverage (UHC) [2], China aims to provide equitable and affordable health care [16, 17]. Since the 1990s, the central government has introduced different health insurance schemes targeting different population groups. In 1998, Urban Employee Basic Medical Insurance (UEBMI) was initiated in urban areas, which is exclusively designed for urban employees [2, 7, 13–15]. Subsequently, another two insurance programs–New Rural Cooperative Medical Scheme (NRCMS) for all rural residents and Urban Resident Basic Medical Insurance (URBMI) for urban residents not eligible for UEBMI (i.e., children under 6 years old, school students, and the unemployed)–were launched in 2003 and 2007, respectively [2, 7, 13–15]. UEBMI, URBMI, and NRCMS constitute basic social health insurance schemes in China. With their introduction, China has successfully achieved UHC by 2020, where more than 97% of the population is insured [18]. However, these three schemes are separately administered at the national level and operated both nationally and locally [16], and vary significantly in terms of premium contributions, health service coverage, benefits packages, and reimbursement rates [3, 13, 15, 19]. Compared to URBMI and NRCMS, UEBMI has more comprehensive health service coverage and stronger financial protection [16, 17]. The fragmentation of social health insurance schemes is the major barrier to health equity in China [14–16, 20]. Hence, since January 2016, integrating NRCMS and URBMI into Urban and Rural Resident Medical Insurance (URRMI) has been rolled out in phases nationwide [16]. This integration reform has played an important role in reducing existing disparities in healthcare access between different insurance plans and increasing the overall healthcare utilization rates [21]. During the period of 2010–2018, the percentage of patients who utilized outpatient and inpatient services increased by 58.6% and 79.6%, respectively [10].

Despite this, disparities in healthcare utilization remain a serious concern in China [17, 22], largely due to population ageing and epidemiological transition [5, 13, 23–26]. Rapid population ageing leads to a growing prevalence of non-communicable diseases (NCDs) [27–30]. NCDs account for 90% of all-cause mortality and 85% of total disease burden in China [31]. The presence of NCDs may result in greater needs for long-term medical treatment, due to their chronic nature [32]. Thus, the middle-aged and elderly should, in theory, utilize more health services to meet their demands [11, 14, 19, 30]. Nonetheless, they are often less likely to access health services [3, 5, 19, 26, 29], given financial difficulties [3]. Consequently, middle-aged and older adults are more vulnerable to disparities in healthcare utilization [1]. Addressing disparities in health service use, particularly in this population group is of significance in China [33].

In addition to rapid demographic and epidemiological transitions, socioeconomic status (SES) is the most fundamental cause of disparities in health service use [1, 6, 23], but little research has investigated this issue. Most of the evidence is from high-income countries [34–40] such as Germany and Australia and some low- and middle-income countries [41–44] such as Brazil, where SES has been measured variously by income [34, 36, 40, 43, 44], education [34, 39, 42, 43], area deprivation index [37], or occupation [38, 40]. The socioeconomic patterning of healthcare utilization in China tends to be different from that in high-income countries and other low- and middle-income countries, due to great differences in health systems and sociodemographic characteristics [23]. In China, there are no gate-keeping general practitioner system and strict referral system [23], where primary health care is delivered by primary care facilities and hospitals for both emergency and non-emergency services [23]. However, few studies in China have examined the effect of SES on healthcare utilization using various indicators of SES such as wealth [5, 45], income [6, 46–48], health insurance [8, 19, 47–51], or education [23, 26, 47, 52]. Nevertheless, it is evident that disparities in health service use in China are primarily determined by individual’s financial capacity [1, 6, 13]. Per-capita household expenditure is a better proxy for financial capacity in the context of China [3, 11, 52, 53], but has less likely been used in the literature. Also, the relationship between SES and health service use is conflicting–some studies show a positive relationship [5, 8, 23, 26, 45, 47, 48, 51], whereas others reveal no association [5, 6, 19, 52]. Moreover, little is known about whether the impact of SES on health service use differs across morbidity status (i.e., no NCDs, a single NCD, and multimorbidity) [1]. Empirical evidence suggests that SES was related to NCDs [39, 53], and the simultaneous occurrence of more than one NCD had an additive impact on health service use [53]. It is thus expected that the prevalence of multiple NCDs may exaggerate the effect of SES on health service use [1]. Additionally, published studies in China were mostly based on cross-sectional study designs [1–5, 7, 9, 13–15, 19, 20, 23, 29, 45, 47–49, 51], while little evidence was from longitudinal studies [8, 24, 26, 52]. Due to broader socioeconomic changes, population ageing, disease burden changes, and health service development [23], investigating the longitudinal changes in healthcare utilization is warranted.

Using longitudinal data from China, our study aims to: (1) identify the trends in healthcare utilization rates over time among adults aged 45 years and above, (2) explore the impact of per-capita household expenditure on health service use, and (3) investigate if having NCDs plays a significant role in the relationship between per-capita household expenditure and healthcare utilization. In the context of unprecedented population ageing and epidemiological transition, attaining health equity is of particular importance in China [3, 6, 7, 9]. Addressing disparities in health service use may have important implications for achieving health equity [33, 46]. Understanding the longitudinal changes in healthcare utilization over time, its association with household economic status, and whether such relationship varies across morbidity status may help develop more targeted policies to redress socioeconomic disparities in healthcare utilization.

## Methods

### Data source and study population

This research used data from four waves of the China Health and Retirement Longitudinal Study (CHARLS) in 2011, 2013, 2015, and 2018 (available at: https://charls.charlsdata.com/pages/data/111/zh-cn.html). The CHARLS is the first nationally representative longitudinal survey of the middle-aged and older conducted by the National School of Development China (Center for Economic Research) at Peking University [54]. Samples in the CHARLS were selected using a four-stage probability-proportional-to-size sampling technique, stratified by per-capita Gross Domestic Product of urban districts and rural counties [54]. A detailed description of sampling technique of the CHARLS has been reported elsewhere [54].

The CHARLS baseline survey involves 17,708 individual participants in 10,257 households, covering 28 provinces, 150 counties/districts, 450 villages/urban communities [54]. The individual participants involved in the baseline were followed over all the four waves in this study. Consequently, the sample in this study included participants aged 45 years and older who responded to all the waves. A total of 10,922 individuals in 6,953 households were finally included in this research. The mean number of household members was 3.80 in 2011, 5.40 in 2013, 3.30 in 2015, and 2.43 in 2018, respectively.

Ethics approval for this study was granted by the Human Research Ethics Committee at University of Canberra. The CHARLS was approved by the Biomedical Ethics Review Committee of Peking University, and all participants were required to complete written informed consent.

### Variables

This study followed Andersen’s Behavioral Model to identify the predictors of healthcare utilization. Andersen’s Behavioral Model is the most widely acknowledged and used model for analyzing health service use [55]. This model argues that individual’s healthcare-seeking behavior is determined by [55, 56]: (1) enabling factors–the mobilization of individual and community resources to utilize health services that are shaped by broader socio-political environment such as income and health insurance, (2) predisposing factors–individual characteristics that exist prior to the development of health need and illness, including demographics (e.g., age, gender, marital status, etc.) and health beliefs (i.e., attitudes, values, and knowledge in relation to health and health services), and (3) need factors–need for health services either perceived by the individual (i.e., how people view their general health, functional states, and illness symptoms) or evaluated by professionals (i.e., professional assessments and objective measurements of patients’ health status and need for health care).

### Dependent variables

Healthcare utilization in this study was categorized into outpatient and inpatient service use, as the patterns of healthcare utilization in China reportedly vary across the type of health services [5, 14, 19].

Outpatient service use in this research was captured by the number of visits to a public hospital, private hospital, public health center, clinic, or health worker’s/doctor’s practice, or the number of times a patient was visited by a health worker or doctor in the last month, whereas inpatient service use was captured by the number of times a patient was hospitalized in the last year [57]. Healthcare utilization indicators were treated as count outcome variables.

### Independent variables

#### SES indicator

To identify the change in the impact of SES on healthcare utilization over time, SES in this study was measured by per-capita household expenditure.

Per-capita household expenditure was calculated using annual total household non-food expenditure (i.e., total household expenditure minus food expenditure) accounting for equivalent household size. Total household expenditure was constructed from a sequence of questions on expenses incurred in the last year, including food expenditure, purchases of durable goods, household utility bills (vehicle or home repairs, etc.), education and health expenditures, discretionary spending items (entertainment, etc.), fees (taxes, etc.), remittances, and transportation costs [57]. The equivalent household size refers to the number of consumption equivalents in the household [58] and has been widely adopted in the literature [59]. The equivalent household size was calculated as the actual household size to the 0.56 power (i.e., actual household size ^0.56^) [59, 60], as an increase in food expenditure is often less than proportional to an increase in household size. Per-capita household expenditure was then categorized into quintiles (Q1-Q5), where Q1 represents the most socioeconomically disadvantaged and Q5 represents the most socioeconomically advantaged.

#### Morbidity status

Morbidity status in this study was grouped into no morbidity, single morbidity, and multimorbidity. Morbidity status was defined as multimorbidity if having two or more co-existing NCDs [1], while it referred to no morbidity and single morbidity if the overall number of NCDs was equal to 0 and 1, respectively. The number of NCDs for each person was individually counted, the range of which was from 0 to 14.

NCDs in this research included disabilities (i.e., brain damage/intellectual disability, hearing problem, speech impediment, vision problem, and physical disabilities) and other 14 NCDs diagnosed by a doctor (i.e., hypertension, diabetes or high blood sugar, dyslipidemia, stroke, asthma, cancer or malignant tumor, heart disease, lung disease, stomach and other digestive diseases, liver disease, kidney disease, arthritis or rheumatism, emotional problems, and memory-related disease) [57]. Disabilities were also considered as one type of NCDs in this study, as disabilities frequently occur due to the presence of NCDs, can worsen people’s quality of life, and may last for people’s whole life course [61].

#### Covariates

Covariates in this study included [57]: (1) demographics such as age groups (45–54, 55–64, 65–74, or 75^+^), sex (male or female), rurality of residence (urban areas, combination zone between urban and rural areas, or rural areas), marital status (separated, divorced, widowed, or unmarried, or married or partnered), ethnicity (Han or other ethnic groups), educational level (illiterate, primary school and below, secondary school, or college school and above (i.e., Two-/Three-Year College/Associate degree, Four-Year College/Bachelor’s degree, Master’s degree, and Doctoral degree)), employment status (notworking (i.e., unemployed and retired) or working (i.e., agricultural work (i.e., engaged in agricultural work for at least 10 days in the past year), non-farm employed paid by wage, and non-farm self-employed/unpaid help)), and health insurance status (no insurance, UEBMI, Unified Basic Medical Insurance (UBMI) (i.e., URBMI, NRCMS, and URRMI), or others (i.e., government medical insurance, medical aid, private medical insurance: purchased by work unit, private medical insurance: purchased by individual, urban non-employed person’s health insurance, long-term care insurance, and other medical insurance)), (2) survey years (2011, 2013, 2015, or 2018), and (3) health behaviors such as smoking status (non-smoker or current smoker) and alcohol drinking status (non-drinker or current drinker) based on the question “whether the participants smoked/drank in the previous year”. Empirical evidence shows that health behaviors played a role in mediating socioeconomic disparities in health outcomes such as mortality and incident cardiovascular disease [62].

### Statistical analysis

**Fig 1:**
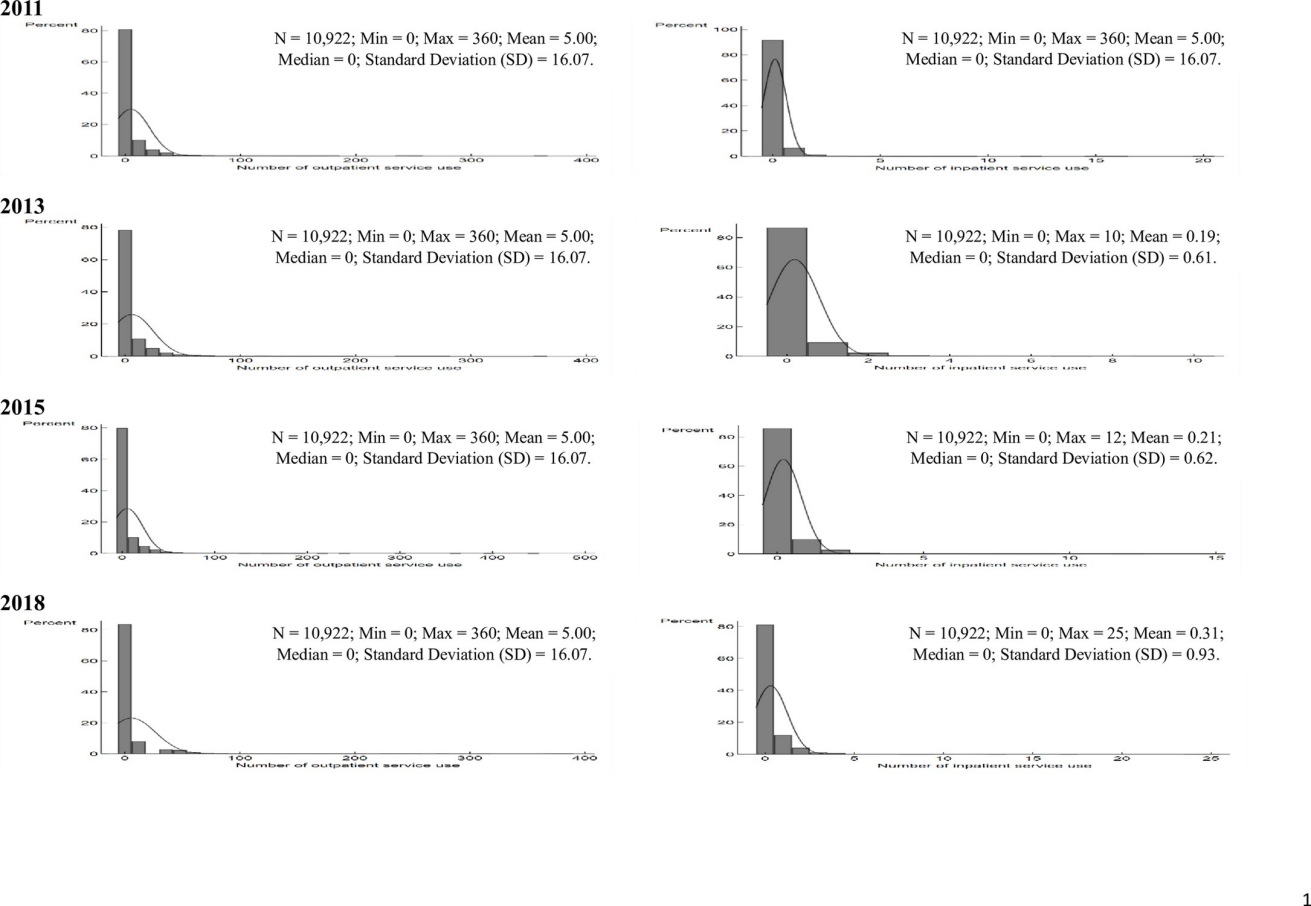
Distribution of the number of outpatient and inpatient service use in 2011, 2013, 2015, and 2018.

Descriptive analysis was performed to describe the study samples using frequencies and percentages. **Fig 1** suggests that healthcare utilization outcomes were non-normally distributed with excessive zeros, regardless of survey years. Consequently, healthcare utilization in this research was treated as an over-dispersed and zero-inflated count variable.

Previous evidence from China suggests that a zero-inflated negative binomial (ZINB) regression model can better address the issue of excessive zeros in healthcare utilization, compared to other zero-inflated models and hurdle models [63]. A ZINB regression model estimates two regression equations: one for modelling counts as the outcome (i.e., count part) and another for modelling an excessive number of zero outcome (i.e., logit part) [63]. Moreover, the sampling frame in the CHARLS includes three levels–individuals, households, and communities, where 10,922 individuals were nested within 6,953 households, which were in turn nested within 442 communities. Individuals within the same household may have the shared traits (e.g., health needs) according to their function and history as a family unit, but there are potentially many differences between households, including living arrangements and household economic conditions [64]. This is also the case for households within the same community and between communities [64]. Using an individual-level regression model may violate the assumption of independent errors and contribute to methodological bias [47, 64]. Hence, this research adopted a four-level ZINB regression model to accommodate the nested effects of household- and community-level determinants.

Self-selection endogeneity is a concern in a growing body of research on health service use [50, 65, 66], as the interaction between individual’s health insurance choices and their healthcare utilization may lead to adverse selection and moral hazard issues [66]. Nonetheless, this may not be the case in the context of China [1, 21]. Under China’s health insurance system, people’s health insurance type depends on household registration systems (urban/rural) and their employment status [21, 65]. Also, the enrolment in UEBMI is mandatory for urban employees [67], while in URBMI and NRCMS, participants are enrolled at the household level to alleviate the adverse selection issue [67]. Furthermore, basic health insurance schemes in China have covered over 97% of the population [18]. It is thus expected that few marked differences exist in the patterns of healthcare utilization between the uninsured and the insured. Additionally, health reforms in China often focus on individual’s responsibility for paying a greater share of their medical costs [68]. High health insurance coverage together with personal responsibility requirements in insurance schemes may maintain reasonable health service use and consequently reduce the occurrence of moral hazard [68]. Therefore, the endogeneity bias is less likely to be a concern in this study.

#### Regression models

Health utilization in this study was initially treated as a binary variable and a logistic regression model was employed to examine statistically significant determinants of healthcare utilization. Results showed that survey years, per-capita household expenditure, morbidity status, age, sex, education, employment, health insurance, smoking status, and drinking status were significantly related to health service use. Accordingly, all these significant predictors were included in the count part of a four-level ZINB regression model for each outcome variable, whereas all these factors except smoking status and drinking status were included in the logit part.

A four-level ZINB regression model was then used for outpatient and inpatient service use, respectively. For each outcome variable, we firstly constructed a null model (Model 1) only with random intercepts in the count part to examine the extent of variance at the individual, household, and community level, respectively. Second, per-capita household expenditure quintiles were added to both count and logit parts of Model 1 (Model 2). After that, morbidity status was added to Model 2 to investigate the effect of per-capita household expenditure quintiles and morbidity status on health service use (Model 3). Finally, other significant predictors were added to Model 3, which was seen as full model (Model 4). The influence of per-capita household expenditure quintiles on health service use did not differ substantially, although such effect improved from Model 2 to Model 4 in terms of Akaike Information Criterion and Bayesian Information Criterion. Thus, only the results of Model 4 were reported for each outcome variable. A possible interaction between morbidity status and per-capita household expenditure quintiles was then added to Model 4 for each outcome variable.

To facilitate the epidemiological interpretation of the results, regression coefficients were exponentiated in both parts of the model–incidence rate ratios (IRR), odds ratios (OR), as well as *p*-value were reported, separately. Any results with a two-sided *p*-value ≤ 0.05 were regarded as statistically significant. All statistical analyses were performed using Stata/SE 16.0 (StataCorp LP, College Station, Texas) and RStudio 2021.09.1+372.

## Results

### Descriptive statistics

There were 10,922 adults included in this study, 52.16% of whom were female. The group aged 55–64 years was the largest (38.83% to 42.27%). The overwhelming majority of people were with Han ethnicity (92.58%), living with a spouse or partner (82.78% to 89.51%), living in rural areas (79.97%), or having educational level of primary school and below (42.70%).

Of 10,922 individuals, 2,087 (19.11%), 2,343 (21.45%), 2,198 (20.12%), and 1,782 (16.32%) utilized outpatient services in 2011, 2013, 2015, and 2018, respectively, while 917 (8.40%), 1,424 (13.04%), 1,548 (14.17%), and 2,052 (18.79%) utilized inpatient services, separately (**Table 1**). From 2011 to 2018, there was a gradually rising trend in the utilization rate of inpatient services (from 8.40% to 18.79%). Nevertheless, the rate of outpatient service use increased from 19.11% in 2011 to 21.45% in 2013 and then reduced to 16.32% in 2018.

The utilization rate of outpatient services was higher in people with multimorbidity (27.28%, 27.15%, 24.54%, and 19.07% in 2011, 2013, 2015, and 2018, respectively), while a majority of individuals who utilized inpatient services were those aged more than 75 years (12.31%, 16.86%, 19.87%, and 27.19%, respectively) or those who were not working (11.41%, 18.25%, 19.35%, and 24.57%, respectively).

**Table 1:** Number (%) of outpatient and inpatient service use in 2011, 2013, 2015, and 2018.

Characteristics	2011		2013		2015		2018	
	Outpatient service use	Inpatient service use	Outpatient service use	Inpatient service use	Outpatient service use	Inpatient service use	Outpatient service use	Inpatient service use
Total	2,087 (19.11)	917 (8.40)	2,343 (21.45)	1,424 (13.04)	2,198 (20.12)	1,548 (14.17)	1,782 (16.32)	2,052 (18.79)
**Demographics**								
Age groups (years)								
45–54	731 (17.88)	257 (6.29)	640 (19.92)	313 (9.74)	501 (19.49)	253 (9.84)	238 (17.59)	185 (13.67)
55–64	846 (19.18)	376 (8.52)	967 (20.94)	617 (13.36)	859 (19.39)	586 (13.23)	690 (16.27)	656 (15.47)
65–74	409 (21.74)	218 (11.59)	554 (23.93)	363 (15.68)	648 (22.58)	500 (17.42)	590 (16.08)	760 (20.71)
75^+^	100 (18.66)	66 (12.31)	182 (23.42)	131 (16.86)	190 (18.06)	209 (19.87)	264 (15.91)	451 (27.19)
Sex								
Male	861 (16.48)	427 (8.17)	966 (18.49)	661 (12.65)	939 (17.97)	718 (13.74)	744 (14.24)	974 (18.64)
Female	1,226 (21.52)	490 (8.60)	1,377 (24.17)	763 (13.39)	1,259 (22.10)	830 (14.57)	1,038 (18.22)	1,078 (18.92)
Rurality of residence								
Urban areas	269 (18.07)	151 (10.14)	326 (21.89)	227 (15.25)	302 (20.28)	246 (16.52)	249 (16.72)	318 (21.36)
Urban-rural areas	134 (19.17)	68 (9.73)	151 (21.60)	98 (14.02)	150 (21.46)	98 (14.02)	128 (18.31)	131 (18.74)
Rural areas	1,684 (19.28)	698 (7.99)	1,866 (21.36)	1,099 (12.58)	1,746 (19.99)	1,204 (13.79)	1,405 (16.09)	1,603 (18.35)
Marital status								
Separated, divorced, widowed, or unmarried	239 (20.86)	100 (8.73)	330 (25.27)	190 (14.55)	316 (21.17)	253 (16.95)	325 (17.28)	429 (22.81)
Married or partnered	1,848 (18.90)	817 (8.36)	2,013 (20.93)	1,234 (12.83)	1,882 (19.96)	1,295 (13.73)	1,457 (16.12)	1,623 (17.95)
Ethnicity								
Han	1,935 (19.14)	830 (8.21)	2,184 (21.60)	1,306 (12.92)	2,042 (20.19)	1,426 (14.10)	1,653 (16.35)	1,881 (18.60)
Other ethnic groups	152 (18.77)	87 (10.74)	159 (19.63)	118 (14.57)	156 (19.26)	122 (15.06)	129 (15.93)	171 (21.11)
Education								
Illiterate	480 (20.03)	205 (8.56)	551 (23.00)	323 (13.48)	494 (20.62)	371 (15.48)	382 (15.94)	490 (20.45)
Primary school and below	950 (20.37)	400 (8.58)	1,041 (22.32)	658 (14.11)	965 (20.69)	684 (14.67)	767 (16.45)	916 (19.64)
Secondary school	626 (16.99)	298 (8.09)	714 (19.38)	424 (11.51)	696 (18.89)	474 (12.86)	596 (16.17)	619 (16.80)
College school and above	31 (17.51)	14 (7.91)	37 (20.90)	19 (10.73)	43 (24.29)	19 (10.73)	37 (20.90)	27 (15.25)
Health insurance								
No insurance	42 (13.73)	12 (3.92)	25 (18.12)	10 (7.25)	19 (15.45)	11 (8.94)	8 (8.33)	10 (10.42)
UEBMI^a^	250 (17.95)	158 (11.34)	313 (21.89)	225 (15.73)	312 (21.41)	254 (17.43)	259 (18.10)	315 (22.01)
UBMI^b^	1,727 (19.35)	713 (7.99)	1,948 (21.44)	1,153 (12.69)	1,835 (19.98)	1,257 (13.69)	1,433 (15.96)	1,644 (18.31)
Others^c^	68 (22.67)	34 (11.33)	57 (21.11)	36 (13.33)	32 (20.00)	26 (16.25)	82 (19.71)	83 (19.95)
Employment status								
Not working	926 (20.97)	504 (11.41)	791 (24.02)	601 (18.25)	717 (20.92)	663 (19.35)	764 (17.44)	1,076 (24.57)
Working	1,161 (17.85)	413 (6.35)	1,552 (20.34)	823 (10.79)	1,481 (19.76)	885 (11.81)	1,018 (15.56)	976 (14.92)
Per-capita household expenditure quintiles^d^								
Q1	304 (15.00)	90 (4.44)	383 (17.99)	177 (8.31)	320 (15.01)	178 (8.35)	311 (14.77)	399 (18.95)
Q2	402 (18.22)	152 (6.89)	456 (20.37)	255 (11.39)	444 (19.87)	270 (12.09)	340 (15.76)	363 (16.82)
Q3	474 (21.41)	176 (7.95)	519 (23.07)	351 (15.60)	522 (22.70)	357 (15.52)	330 (14.99)	401 (18.22)
Q4	450 (20.02)	232 (10.32)	512 (22.85)	368 (16.42)	479 (21.46)	412 (18.46)	389 (17.19)	446 (19.71)
Q5	457 (20.51)	267 (11.98)	473 (22.93)	273 (13.23)	433 (21.39)	331 (16.35)	412 (18.77)	443 (20.18)
**Morbidity status**								
No morbidity	319 (10.07)	121 (3.82)	421 (14.51)	206 (7.10)	276 (13.04)	152 (7.18)	78 (6.20)	60 (4.77)
Single morbidity	520 (16.36)	197 (6.20)	572 (18.76)	318 (10.43)	417 (15.61)	278 (10.40)	196 (11.16)	191 (10.87)
Multimorbidity	1,248 (27.28)	599 (13.09)	1,350 (27.15)	900 (18.10)	1,505 (24.54)	1,118 (18.23)	1,508 (19.07)	1,801 (22.78)
**Health behaviors**								
Smoking status								
Non-smoker	1,359 (20.58)	560 (8.48)	2,074 (22.48)	1,256 (13.61)	1,629 (21.70)	1,146 (15.26)	1,414 (17.62)	1,665 (20.74)
Current smoker	728 (16.85)	357 (8.26)	269 (15.87)	168 (9.91)	569 (16.67)	402 (11.78)	368 (12.71)	387 (13.37)
Alcohol drinking status								
Non-drinker	1,509 (20.88)	670 (9.27)	1,689 (23.72)	1,057 (14.84)	1,534 (21.43)	1,131 (15.80)	1,288 (17.34)	1,558 (20.97)
Current drinker	578 (15.64)	247 (6.68)	654 (17.21)	367 (9.66)	664 (17.64)	417 (11.08)	494 (14.14)	494 (14.14)

Note.

^a^ UEBMI = Urban Employee Basic Medical Insurance

^b^ UBMI = Unified Basic Medical Insurance

^c^ Others = Government medical insurance, Medical aid, Private medical insurance: purchased by work unit, Private medical insurance: purchased by individual, urban non-employed person’s health insurance, Long-term care insurance, and Other medical insurance

^d^ Q1 is the poorest and Q5 is the wealthiest.

A four-level ZINB regression model separately for outpatient and inpatient service use.

Significant differences were found in the probability of outpatient service use across per-capita household expenditure quintiles and morbidity status (**Table 2**). Compared to those in the lowest quintile of per-capita household expenditure (Q1), individuals in Q2 (OR = 1.233, *p* < 0.0001), Q3 (OR = 1.416, *p* < 0.0001), Q4 (OR = 1.408, *p* < 0.0001), or Q5 (OR = 1.439, *p* < 0.0001) were more likely to utilize outpatient services. Likewise, compared to those with no morbidity, adults having single morbidity (OR = 1.467, *p* < 0.0001) or multimorbidity (OR = 2.571, *p* < 0.0001) were more likely to utilize outpatient services. While the count part of the model revealed that, higher rates of outpatient service use were observed for individuals in Q4 (IRR = 1.089, *p* = 0.002) and Q5 (IRR = 1.072, *p* = 0.011) (compared to those in Q1) and those with multimorbidity (IRR = 1.187, *p* < 0.0001) (compared to those with no morbidity).

**Table 2:** A four-level zero-inflated negative binomial regression model for outpatient service use^a^.

Characteristics	Count part		Logit part	
	IRR for the number of healthcare utilization	*P*-value	OR for having healthcare utilization	*P*-value
**Survey years**				
2011				
2013	**1.055**	**0.014**	**1.136**	**0.000**
2015	1.018	0.405	0.962	0.270
2018	**1.252**	**< 0.0001**	**0.656**	**< 0.0001**
**Per-capita household expenditure quintiles** ^**b**^				
Q1				
Q2	1.021	0.442	**1.233**	**< 0.0001**
Q3	1.048	0.078	**1.416**	**< 0.0001**
Q4	**1.089**	**0.002**	**1.408**	**< 0.0001**
Q5	**1.072**	**0.011**	**1.439**	**< 0.0001**
**Morbidity status**				
No morbidity				
Single morbidity	1.038	0.214	**1.467**	**< 0.0001**
Multimorbidity	**1.187**	**< 0.0001**	**2.571**	**< 0.0001**
**Age groups (years)**				
45–54				
55–64	**1.053**	**0.025**	0.975	0.445
65–74	1.041	0.146	1.027	0.507
75^+^	1.067	0.096	0.855	0.006
**Sex**				
Male				
Female	**1.046**	**0.050**	**1.163**	**< 0.0001**
**Education**				
Illiterate				
Primary school and below	0.966	0.167	1.004	0.912
Secondary school	**0.935**	**0.025**	**0.930**	**0.042**
College school and above	0.928	0.342	1.004	0.971
**Health insurance**				
No insurance				
UEBMI^c^	0.855	0.055	1.259	0.055
UBMI^d^	0.885	0.106	**1.303**	**0.020**
Others^e^	0.999	0.990	**1.440**	**0.007**
**Employment status**				
Not working				
Working	0.990	0.623	**0.943**	**0.032**
**Smoking status**				
Non-smoker				
Current smoker	1.016	0.510	n.p.^f^	n.p.^f^
**Alcohol drinking status**				
Non-drinker				
Current drinker	**0.955**	**0.026**	n.p.^f^	n.p.^f^
**Variance components**	**Variance**		**Standard deviation**	
Individual	0.137		0.371	
Household	0.047		0.217	
Community	0.016		0.125	
**Akaike Information Criterion (AIC)**	111,856.5			
**Bayesian Information Criterion (BIC)**	112,238.6			

Note.

^a^ Incidence rate ratios (IRR), Odds ratios (OR), and *p*-value significantly related to healthcare utilization are bolded

^b^ Q1 is the poorest and Q5 is the wealthiest

^c^ UEBMI = Urban Employee Basic Medical Insurance

^d^ UBMI = Unified Basic Medical Insurance

^e^ Others = Government medical insurance, Medical aid, Private medical insurance: purchased by work unit, Private medical insurance: purchased by individual, urban non-employed person’s health insurance, Long-term care insurance, and Other medical insurance

^f^ n.p. = not reported.

Similarly, individuals with single morbidity (OR = 2.690, *p* = 0.015) or multimorbidity (OR = 8.926, *p* < 0.0001) were more likely to utilize inpatient services, compared to those with no morbidity (**Table 3**). However, there was no significant difference in the probability of inpatient service use across per-capita household expenditure quintiles. While the count part of the model suggested that, higher rates of inpatient care utilization were observed for those in Q2 (IRR = 1.273, *p* < 0.0001), Q3 (IRR = 1.773, *p* < 0.0001), Q4 (IRR = 2.071, *p* < 0.0001), or Q5 (IRR = 1.992, *p* < 0.0001) (compared to those in Q1) and those with single morbidity (IRR = 1.504, *p* < 0.0001) or multimorbidity (IRR = 2.558, *p* < 0.0001) (compared to those with no morbidity). Likewise, individuals aged 55–64 years (IRR = 1.160, *p* = 0.001), those aged 65–74 years (IRR = 1.314, *p* < 0.0001), or those aged over 75 years (IRR = 1.480, *p* < 0.0001) had higher rates of inpatient service use, compared to those aged 45–54 years. On the contrary, lower rates of inpatient service use were observed for current smokers (IRR = 0.739, *p* < 0.0001) and current drinkers (IRR = 0.700, *p* < 0.0001), compared to non-smokers and non-drinkers.

**Table 3:** A four-level zero-inflated negative binomial regression model for inpatient service use^a^.

Characteristics	Count part		Logit part	
	IRR for the number of healthcare utilization	*P*-value	OR for having healthcare utilization	*P*-value
**Survey years**				
2011				
2013	**1.531**	**< 0.0001**	3.241	0.589
2015	**1.553**	**< 0.0001**	*^b^	0.998
2018	**2.258**	**< 0.0001**	**0.141**	**0.008**
**Per-capita household expenditure quintiles** ^**c**^				
Q1				
Q2	**1.273**	**< 0.0001**	*^b^	1.000
Q3	**1.773**	**< 0.0001**	*^b^	1.000
Q4	**2.071**	**< 0.0001**	*^b^	1.000
Q5	**1.992**	**< 0.0001**	*^b^	1.000
**Morbidity status**				
No morbidity				
Single morbidity	**1.504**	**< 0.0001**	**2.690**	**0.015**
Multimorbidity	**2.558**	**< 0.0001**	**8.926**	**< 0.0001**
**Age groups (years)**				
45–54				
55–64	**1.160**	**0.001**	0.732	0.443
65–74	**1.314**	**< 0.0001**	1.138	0.794
75^+^	**1.480**	**< 0.0001**	3.336	0.148
**Sex**				
Male				
Female	**0.730**	**< 0.0001**	0.731	0.380
**Education**				
Illiterate				
Primary school and below	0.969	0.543	1.221	0.605
Secondary school	**0.881**	**0.036**	1.006	0.987
College school and above	**0.595**	**0.003**	0.821	0.878
**Health insurance**				
No insurance				
UEBMI^d^	1.405	0.082	**8.829**	**0.043**
UBMI^e^	**1.442**	**0.048**	3.347	0.179
Others^f^	**1.627**	**0.020**	5.801	0.155
**Employment status**				
Not working				
Working	**0.654**	**< 0.0001**	1.222	0.533
**Smoking status**				
Non-smoker				
Current smoker	**0.739**	**< 0.0001**	n.p.^g^	n.p.^g^
**Alcohol drinking status**				
Non-drinker				
Current drinker	**0.700**	**< 0.0001**	n.p.^g^	n.p.^g^
**Variance components**	**Variance**		**Standard deviation**	
Individual	0.697		0.835	
Household	0.208		0.456	
Community	0.066		0.256	
**Akaike Information Criterion (AIC)**	42,484.3			
**Bayesian Information Criterion (BIC)**	42,866.5			

Note.

^a^ Incidence rate ratios (IRR), Odds ratios (OR), and *p*-value significantly related to healthcare utilization are bolded

^b^ The value of OR is extremely small or large and was not reported here

^c^ Q1 is the poorest and Q5 is the wealthiest

^d^ UEBMI = Urban Employee Basic Medical Insurance

^e^ UBMI = Unified Basic Medical Insurance

^f^ Others = Government medical insurance, Medical aid, Private medical insurance: purchased by work unit, Private medical insurance: purchased by individual, urban non-employed person’s health insurance, Long-term care insurance, and Other medical insurance

^g^ n.p. = not reported.

Interaction between morbidity status and per-capita household expenditure quintiles.

Morbidity status did not play a significant role in moderating the relationship between per-capita household expenditure quintiles and healthcare utilization, irrespective of the type of health services (**Table 4**).

**Table 4:** Interaction between morbidity status and per-capita household expenditure quintiles for outpatient and inpatient service use^a^^,^^b^.

Characteristics	Outpatient service use		Inpatient service use		
	IRR for the number of healthcare utilization (*P*-value)	OR for having healthcare utilization (*P*-value)	IRR for the number of healthcare utilization (*P*-value)	OR for having healthcare utilization (*P*-value)	
**Per-capita household expenditure quintiles** ^**c**^					
Q1					
Q2	0.987 (0.859)	1.137 (0.248)	1.370 (0.338)	*^d^ (0.995)	
Q3	1.059 (0.434)	**1.468 (0.000)**	1.499 (0.216)	*^d^ (0.982)	
Q4	1.080 (0.304)	**1.357 (0.004)**	1.506 (0.213)	*^d^ (0.998)	
Q5	1.053 (0.492)	**1.392 (0.002)**	**2.631 (0.005)**	0.335 (0.712)	
**Morbidity status**					
No morbidity					
Single morbidity	1.020 (0.783)	**1.482 (0.000)**	**2.241 (0.013)**	0.156 (0.512)	
Multimorbidity	**1.177 (0.009)**	**2.476 (< 0.0001)**	**3.996 (< 0.0001)**	0.228 (0.599)	
Morbidity status * Per-capita household expenditure quintiles ^**c**^					
No morbidity * Q1					
Single morbidity * Q2	1.060 (0.548)	1.066 (0.652)	0.550 (0.086)	37.902 (1.000)
Single morbidity * Q3	1.034 (0.718)	0.930 (0.591)	0.795 (0.518)	*^d^ (0.986)
Single morbidity * Q4	0.976 (0.798)	0.985 (0.914)	0.928 (0.828)	56.149 (1.000)
Single morbidity * Q5	1.019 (0.840)	0.987 (0.927)	0.505 (0.059)	*^d^ (0.999)
Multimorbidity * Q2	1.033 (0.693)	1.111 (0.388)	0.645 (0.177)	0.306 (1.000)
Multimorbidity * Q3	0.975 (0.748)	0.968 (0.783)	0.769 (0.416)	*^d^ (1.000)
Multimorbidity * Q4	1.020 (0.805)	1.064 (0.600)	0.916 (0.786)	24.386 (1.000)
Multimorbidity * Q5	1.021 (0.800)	1.057 (0.640)	0.532 (0.058)	9.915 (0.416)

Note.

^a^ Models were fitted with main effects, interaction, and adjusted for covariates, but for space reasons, only per-capita household expenditure quintiles, morbidity status, and interaction terms were reported here

^b^ Incidence rate ratios (IRR), odds ratios (OR), and *p*-value significantly related to healthcare utilization are bolded

^c^ Q1 is the poorest and Q5 is the wealthiest

^d^ Due to small sample size for certain category, the value of OR is extremely small or large and was not reported here.

## Discussion

Using the nationally representative CHARLS data collected in 2011, 2013, 2015, and 2018, this study found an increasing trend in outpatient service use from 2011 to 2013 and then a decreasing trend, and a gradually rising trend in inpatient service use, but healthcare utilization rates were overall low. This study also showed that higher quintiles of per-capita household expenditure were significantly related to higher odds of outpatient service use and higher rates of inpatient service use. Additionally, this research revealed that having morbidity was generally associated with increased healthcare utilization but did not play a significant role in moderating the effect of SES on health service use.

This study found that only a small proportion of the study population utilized outpatient (19.11% in 2011, 21.45% in 2013, 20.12% in 2015, and 16.32% in 2018, respectively) or inpatient (8.40%, 13.04%, 14.17%, and 18.79%, separately) services. These findings are comparable to those from previous studies in China, where the utilization rates of outpatient and inpatient services were 18.3% [13] or 18.6% to 20.7% [8], and 9.6% to 14.3% [8], 13.7% [13], or 17.7% [5], respectively. Healthcare utilization rates in China are lower than those in high-income countries (20.3% [37], 37.5% [37], or even 78% [36]). This is possibly because low affordability is the major obstacle to accessing health services [6, 13]. The financial burden of health expenditure has substantially increased in China over the recent decades [13], given sharp increases in medical costs and the share of out-of-pocket payments to total health expenditure [13]. Another explanation for low healthcare utilization rates could be that self-medication is a potential option for health care, due to higher medical costs and limited access to health care [23].

This research also suggested a gradually growing trend in the utilization rate of inpatient services between 2011 and 2018, but the rate of outpatient service use rose from 19.11% in 2011 to 21.45% in 2013 and then decreased to 16.32% in 2018. From 2011 to 2018, inpatient utilization rate increased by 2.24 times (from 8.4% to 18.79%), which is consistent with the evidence that hospitalization rates in China more than doubled over the past decade [22, 46]. However, this trend contrasts with the overall goal of health reforms in China, which emphasizes the utilization of primary health care services [16]. This is possibly because the reimbursement policies focus more on inpatient services [2, 69]. Therefore, there is a need to further optimize health insurance schemes, especially for outpatient services [5], which may encourage the use of outpatient services for health promotion and prevention rather than seeking inpatient care.

Furthermore, this study showed that higher quintiles of per-capita household expenditure were significantly related to higher odds of outpatient service use and higher rates of inpatient service use, which consolidates the empirical evidence that health service use often favored the better-off [5, 8, 23, 26, 45, 47, 48, 51]. The potential explanation could be that individuals in higher quintiles can better articulate their demands for regular health care [5]. Nevertheless, this research failed to find a significant relationship between per-capita household expenditure quintiles and the probability of inpatient service use, which consolidates a prior study in China [5]. Overall, consistent with prior research in China [5, 45], our finding may indicate great disparities in whether to access outpatient services and the number of inpatient service use among different socioeconomic groups of people aged over 45 years who require regular health care. As patient’s willingness to access health services mainly depends on reimbursement rates of health insurance plans [5, 13], further optimizing health insurance schemes is needed, particularly for outpatient services [5]. Disparities in healthcare utilization remain a crucial concern in China [17, 22]; therefore, further examining the underlying causes is warranted, considering both urban-rural segmentations and constraints (e.g., health out-of-pocket payment) [23]. It is also worth exploring the cross-country comparisons on the patterns of health service use, which may deepen our understanding of different socioeconomic, policy, and cultural contexts of population ageing [23].

Moreover, this research found that having morbidity was generally associated with higher healthcare utilization, which consolidates previous findings [23, 53, 70]. NCDs often impair people’s quality of life and their functional ability [70]; thus, patients with NCDs often increase health service use to meet their health needs [32, 53, 70]. Given rapid population ageing and increasing burden of NCDs [5, 13, 23, 24], disparities in health service use tend to pose a more significant threat to China’s health system [19]. Hence, new health service delivery models to effectively manage NCDs are needed [70]. Nonetheless, this research failed to find that morbidity status played a significant role in moderating the relationship between per-capita household expenditure quintiles and health service use. Likewise, earlier evidence from the 2011–2015 CHARLS data suggested a similar magnitude of the effect of an additional NCD on healthcare utilization across per-capita household expenditure quintiles [53]. People in higher quintiles are more likely to have better health literacy and more access to health services [53]; therefore, they are less likely to have NCDs or have NCDs diagnosed at an earlier stage. In contrast, those in lower quintiles tend to less utilize health services; thus, they do not often have NCDs diagnosed. Our findings may embody a combination of the prevalence of NCDs, health literacy, and access to health care in the context of China [53]. Given higher non-utilization rates of health services in China, it is necessary to further investigate the underlying reasons why morbidity status, despite significantly related to healthcare utilization, did not play a role in the effect of per-capita household expenditure quintiles on health service use.

In addition, this study suggested that advanced age was significantly associated with higher rate of inpatient service use, which is consistent with previous studies in China [5, 23, 49]. Population ageing undoubtedly contributes to more health demands [27, 28], and as the population ages, the growing prevalence of NCDs [19, 30] may also significantly increase their demands for health services [32]. On the contrary, this study showed that healthcare utilization rates were generally lower for current smokers and current drinkers than non-smokers and non-drinkers, which corroborates prior evidence from China [23]. This finding may mirror the Chinese cultural and policy context–as people get older, they may gradually develop into a habit of healthier lifestyles [23]. Stopping smoking or drinking after having health problems tends to be an adaptive healthy behavior of the elderly in China [23].

Providing equitable and affordable health care for all is at the core of China’s health system [1–3, 16, 17]. To achieve this goal, future health policies should take accessibility, affordability, and availability of health services into account [23]. Given pro-rich disparities in healthcare utilization, more attention should be given to pro-poor financing strategies [6]. It is necessary to further optimize health insurance schemes targeting outpatient services, particularly for those with low SES [6, 71]. Specifically, expanding health service coverage, increasing reimbursement ratios, decreasing deductibles, and optimizing co-payment are needed [23]. Other demand-side measures such as more financial assistance to the low-SES may also play a role. While supply-side interventions should target primary health care. China’s health system is hospital-centred and thus causes massive cost pressures on hospitals [23, 30]. The experience from western countries indicates that service provision shifting to primary care facilities may improve equity in health service use [23] and having NCDs treated at these centers is more cost-effective [7, 23]. Therefore, it is imperative to establish new health delivery models to effectively manage NCDs in the primary health care setting [6, 23, 49, 72].

### Limitations

This study is subject to some limitations; therefore, results should be interpreted with caution. First, healthcare utilization in this study was measured by few simple questions in the CHARLS, which only captured the number of health service use [1]. Further studies require examining healthcare utilization based on indicators in relation to the quality of health services and financial burden [1]. Second, one-year recall period for health service use may result in recall bias and not necessarily fully captures people’s actual outpatient and inpatient care utilization. Lastly, self-reported measures of health status are prone to measurement errors than clinical assessments [4, 23]. The accuracy of self-reported health status relies on individual’s health awareness level [4]; hence, under- or over-estimation of healthcare utilization may occur between different SES groups [4].

Despite these limitations, all the findings from this study may inform health policies to provide more equitable and affordable health services in China, thereby achieving health equity as proposed in the Sustainable Development Goals and the “Healthy China 2030” Plan.

## Conclusions

Using the CHARLS data from the 2011, 2013, 2015, and 2018 survey waves, this research found a growing trend in outpatient care utilization from 2011 to 2013 and then a decreasing trend, and a gradually increasing trend in inpatient care utilization, but healthcare utilization rates were overall low. This study also showed positive associations of per-capita household expenditure quintiles with the probability of outpatient service use and the count of inpatient service use. Additionally, this research suggested that having morbidity generally increased health service use but did not play a significant role in moderating the relationship between SES and healthcare utilization. To deliver more equitable health care, future health policies in China should focus on providing more health education to the public, further optimizing health insurance schemes targeting outpatient services, especially for the low-SES, and establishing new health delivery models for NCD management in the primary health care setting.

## Supporting information

S1 Checklist STROBE statement—checklist of items that should be included in reports of observational studies. (DOCX)
